# Blue Light Irradiation Induces Human Keratinocyte Cell Damage via Transient Receptor Potential Vanilloid 1 (TRPV1) Regulation

**DOI:** 10.1155/2020/8871745

**Published:** 2020-12-15

**Authors:** Ju Ah. Yoo, Eunbi Yu, See-Hyoung Park, Sae Woong Oh, Kitae Kwon, Se Jung Park, Hyeyoun Kim, Seyoung Yang, Jung Yoen Park, Jae Youl Cho, Youn-Jung Kim, Jongsung Lee

**Affiliations:** ^1^Molecular Dermatology Laboratory, Department of Integrative Biotechnology, College of Biotechnology and Bioengineering, Sungkyunkwan University, Suwon City, 16419 Gyunggi Do, Republic of Korea; ^2^Department of Bio and Chemical Engineering, Hongik University, 30016 Sejong City, Republic of Korea; ^3^Molecular Immunology Laboratory, Department of Integrative Biotechnology, College of Biotechnology and Bioengineering, Sungkyunkwan University, Suwon City, 16419 Gyunggi Do, Republic of Korea; ^4^Department of Marine Sciences, Incheon National University, 22012 Incheon City, Republic of Korea

## Abstract

Although blue light has been reported to affect skin cells negatively, little is known about its action mechanisms in skin cells. Therefore, we investigated the role of the transient receptor potential vanilloid 1 (TRPV1) in blue light-induced effects on human keratinocytes and its underlying mechanisms. Blue light decreased cell proliferation and upregulated TRPV1 expression. Blue light also suppressed the epidermal growth factor receptor- (EGFR-) mediated signaling pathway by reducing the protein levels of EGFR and suppressing the EGFR/PI3K/AKT/GSK3*β*/FoxO3a pathway. The blue light-induced effect in cell proliferation was reversed by TRPV1 siRNA, but not capsazepine, a TRPV1-specific antagonist. In addition, blue light irradiation increased the production of reactive oxygen species (ROS) and tumor necrosis factor-*α* (TNF-*α*). Blue light irradiation also increased both phosphorylation levels of TRPV1 and calcium influx. The blue light-induced increase in production of ROS and TNF-*α* was reversed by capsazepine. Furthermore, the blue light-induced increase in production of TNF-*α* was attenuated by SP600125 or PDTC. These findings show that blue light regulates cell survival and production of ROS and TNF-*α*; its effects are mediated via TRPV1. Specifically, the effects of blue light on cell proliferation are mediated by upregulating TRPV1, a negative regulator of EGFR-FoxO3a signaling. Blue light-induced production of ROS and TNF-*α* is also mediated through increased calcium influx via TRPV1 activation.

## 1. Introduction

The skin is an important organ that protects the human body against external stimuli, acting as an essential barrier [1]. It detects harmful stresses such as sunlight, chemicals, or bacteria; induces an inflammatory response to damage; and maintains transdermal moisture [2]. These functions are maintained by skin homeostasis [3].

Skin homeostasis is regulated by the proliferation, differentiation, and apoptosis of epidermal keratinocytes [4]. Keratinocytes proliferate at the stratum basale, the innermost layer of the epidermis, and begin to differentiate as they rise to the upper layer [3]. When they reach the outermost layer, they are terminally differentiated into corneocytes, which are dead keratinocytes containing filamentous keratin, forming a stratum corneum [5]. The stratum corneum functions as a skin barrier, and its corneocytes are desquamated from the body [5]. This process is repeated at regular intervals; thus, the skin barrier can be regenerated and maintained [2, 3, 5, 6]. Depending on the position of each layer of the skin, various differentiation processes occur due to the gradient of the calcium ion concentration in each layer [7, 8]. Therefore, factors influencing the calcium ion influx in keratinocytes may affect the skin barrier homeostasis.

TRPV1, a member of the transient receptor potential (TRP) cation channel family, is expressed in kidney cells, bronchial epithelial cells, primary sensory neurons, and keratinocytes [9, 10]. TRPV1 is a nonselective cation receptor and responds to capsaicin, which is widely used as a pungent spice [11, 12]. TRPV1 is also activated by high temperatures (>42°C), low pHs, and ultraviolet (UV) light [10, 13–15]. When activated, TRPV1 induces calcium ion influx into keratinocytes and mediates multiple signaling pathways [9, 10, 16]. Generally, it inhibits cell proliferation, promotes differentiation, and induces the secretion of several proinflammatory cytokines in response to the above stimuli [9, 10, 13, 17, 18]. In addition, TRPV1 has been known to be involved in skin aging via the induction of collagen degradation, which occurs through the increase in MMP-1 expression, in the HaCaT cell line [19]. On the other hand, in synoviocytes, an increase in the cytosolic calcium ion concentration via TRPV1 activation is known to cause ROS production, thus leading to cell death [20]. These reports suggest that TRPV1 has similar effects on the skin. In addition, the expression of TRPV1 has been recently reported to induce the degradation of the epidermal growth factor receptor (EGFR), inhibiting skin carcinogenesis and cellular metabolism. AMG9810, an antagonist of TRPV1, was shown to cause skin tumorigenesis through EGFR/AKT signaling [21, 22].

Blue light is a part of the visible light spectrum, with wavelengths ranging from 400 to 500 nm; its negative effects on retinal cells and the prevention of these effects have been studied [23–25]. While the effects of UV light on the skin have been studied continuously, there has not been much research on the effects of visible light, which accounts for a large proportion of sunlight [14, 26, 27]. Recent studies have shown that blue light inhibits the proliferation of skin keratinocytes and induces ROS production [28, 29]. However, the mechanisms underlying its action have not yet been demonstrated clearly. Therefore, in this study, we investigated the effects of blue light on keratinocytes and the mechanisms underlying these effects. Specifically, we examined the involvement of TRPV1 in the effects of blue light on keratinocytes and the related signaling mechanisms.

## 2. Materials and Methods

### 2.1. Cell Culture

HaCaT cells, a human keratinocyte cell line, were cultured in Dulbecco's modified Eagle's medium (DMEM) supplemented with 10% fetal bovine serum (FBS) and 1% antibiotics (penicillin/streptomycin) in a humidified atmosphere containing 5% CO_2_ at 37°C. The HEK293-TRPV1-luciferase stable cell line was maintained in DMEM supplemented with 10% FBS, 1% antibiotics, and 10% puromycin in a humidified atmosphere containing 5% CO_2_ at 37°C.

### 2.2. Blue Light Irradiation

First, the cells were plated on 60*π* dishes; after one day, they were irradiated using an LED emitting blue light with a wavelength of 470–480 nm, at a power density of 76 W/m^2^. The cells were irradiated at room temperature for 10 min (45.6 mJ/cm^2^), 15 min (68.4 mJ/cm^2^), and 30 min (136.8 mJ/cm^2^) in a photoreactor (CCP-4V, Luzchem). The doses used are equivalent to those received by the skin during summer after proximately 15 seconds of sun exposure. Before irradiation, the cultured dishes were rinsed once with transparent media and irradiated in phenol red-free media to avoid the absorption of irradiation by colored media. To establish equal conditions, cells in the control group were also maintained in phenol red-free media at room temperature while those in the experimental groups were irradiated.

### 2.3. Cell Viability Assay

The effect of blue light with wavelengths of 470–480 nm on the viability of HaCaT cells was determined using cell counting kit-8 assay (CCK-8, Dojindo, Japan). HaCaT cells cultured in 60*π* cell plates were irradiated with blue light for various time periods on three successive days. The CCK-8 reagent (8 *μ*L/well) was added to each well, and the plates were incubated at 37°C for a further 2 h on day 4. The supernatants were added into 96-well plates, and their absorbances were measured at 450 nm using a microplate reader (Synergy HTX Multi-Mode Reader, BioTek, VT, USA).

### 2.4. Small Interfering RNA (siRNA) Transfection

ON-TARGETplus SMARTpool human TRPV1 siRNA (L-006518-00-0020) and ON-TARGETplus Non-targeting pool (D-001810-10-20) were synthesized by Dharmacon Research (Lafayette, CO, USA). The cells were transfected with 25 nM of the indicated siRNAs for 48 h using the DharmaFECT transfection agent (Dharmacon Research), according to the manufacturer's instructions.

### 2.5. Fluo-4 Calcium Influx Assay

Cells were cultured in 96-well black-wall/white-bottom microplates to near confluence. Next, 2x Fluo-4 Direct™ calcium reagent loading solution (F10471, Invitrogen) was added directly to the wells containing the cells in the culture medium. The plates were incubated at 37°C for 30 minutes and at room temperature for 30 minutes. All loading processes were performed under conditions of light blocking. After the loading of the Fluo-4 Direct reagent, the cells were stimulated with blue light or the agonist capsaicin (Sigma). In the case of the experimental groups, the cells were treated with the indicated concentration of capsazepine (Sigma) 30 minutes before their irradiation. Fluorescence was measured using a microplate reader at excitation and emission wavelengths of 494 nm and 516 nm, respectively.

### 2.6. DCFDA-Cellular Reactive Oxygen Species (ROS) Detection Assay

ROS production was quantitatively measured using the DCFDA-cellular reactive oxygen species (ROS) detection assay kit (ab113851) and analyzed using fluorescence microscopy and a microplate reader. The cells were seeded onto 60*π* dishes or 96-well plates. The cultured cells were irradiated with blue light or TBHP solution, which was used as a positive control. After 24 hours, they were washed twice in PBS and stained with 25 *μ*M DCFDA in PBS for 15 min at 37°C in the dark. The stained cells were washed, and their fluorescence signals were detected at an Ex/Em of 485/535 nm. The change in fluorescence was determined as the percentage of the control fluorescence after background subtraction.

### 2.7. Extraction of the Nuclear and Cytoplasmic Fractions

The nuclear fractions were isolated to confirm the translocation of transcription factors using Western blotting. NE-PER Nuclear and Cytoplasmic Extraction Reagents (78833, Thermo Fisher Scientific) were used, and the protocol recommended by the manufacturer was followed.

### 2.8. Western Blotting Analysis

HaCaT cells were seeded onto 60*π* cell plates. The cells were harvested and centrifuged for 5 min at 13,000 rpm. The supernatant was discarded, and the cells were lysed with the RIPA lysis buffer (25 mM Tris-HCl (pH 7.6), 150 mM NaCl, 1% NP-40, 1% sodium deoxycholate, and 0.1% SDS (Thermo Fisher Scientific, Waltham, MA, USA)) containing Halt protease and a phosphatase inhibitor cocktail (Thermo Fisher Scientific). The proteins extracted from the cells were separated by 8–10% SDS electrophoresis and transferred onto nylon membranes. The membranes were blocked with 5% skim milk for 1 h and then incubated with primary antibodies at 4°C overnight. The membranes were washed thrice with Tris-buffered saline (TBS) containing Tween 20 and probed with secondary antibodies for 1 h at room temperature. The blots were visualized using ECL Western Blotting Reagents.

### 2.9. Analysis of mRNA Levels Using Real-Time RT-PCR

Real-time RT-PCR analysis was performed using an ABI7900HT Instrument (Applied Biosystems, Waltham, MA, USA). For TaqMan analysis, predesigned or optimized assays on demand (Applied Biosystems) were used, including EGFR (ID: Hs01076090_m1), TRPV1 (ID: Hs00949993_g1), glyceraldehyde-3-phosphate dehydrogenase (GAPDH) (ID: Hs00266705_g1), hypoxanthine-guanine phosphoribosyltransferase (HPRT) (Hs02800695_m1), and 18S (Hs03003631_g1). The data were analyzed using ABI Sequence Detector Software version 2.0 (Applied Biosystems). Total RNA was extracted from cells using the TRI reagent® according to the manufacturer's instructions and stored at -70°C until use. cDNA was synthesized from total RNA (1 *μ*g) using MuLV reverse transcriptase according to the manufacturer's instructions. Real-time RT-PCR analysis was conducted as previously described [30]. The results were normalized to the expression level of endogenous GAPDH and were also tested against two additional housekeeping genes (18S and HPRT). We found that the results were not significantly different from those obtained using GAPDH. Expression levels of target genes were normalized to the levels observed in controls. Results were verified through four-time repetition of the same experiment, each of which was conducted in triplicate.

### 2.10. Reporter Luciferase Assay

Luciferase reporter assay and *β*-galactosidase assay were performed to determine the promoter activities at the transcriptional level. NF-*κ*B (Stratagene, La Jolla, CA, USA), CRE (Stratagene), AP-1 (Stratagene), and FoxO3a (Addgene, MA, USA) promoter-firefly luciferase reporters were used. Cells were seeded onto 60*π* cell plates and incubated at 37°C overnight. The cells were cotransfected with 1.5 *μ*g of the promoter-luciferase reporters and 1.5 *μ*g of the *β*-galactosidase reporter vector (Promega Corporation) using 7.5 *μ*g polyethylenimine (Sigma-Aldrich, St. Louis, MO, USA) to quantify the promoter-luciferase activities. Four hours after the transfection, the cells were cultured in a new medium for 24 h and then irradiated with blue light on day 2. After 24 h of incubation at 37°C, *β*-galactosidase activity was assayed using the *β*-Galactosidase Enzyme Assay System with the Reporter Lysis Buffer (Promega Corporation). The cells were harvested with PBS and lysed with the Reporter Lysis Buffer (Promega Corporation). The cells were then centrifuged, and the supernatants were transferred into 96-well plates to quantify the *β*-galactosidase activity. Color development was stopped by the addition of 1 M sodium carbonate to the wells, and the absorbance of the samples was measured at 420 nm using a microplate reader to quantify the *β*-galactosidase activity. Luciferase activity was assayed using the Luciferase Activity Assay System (Promega Corporation). The cells were harvested with PBS and lysed with the Reporter Lysis Buffer (Promega Corporation). The cells were then centrifuged, and the supernatants were transferred into 96-well plates. The luciferase assay substrate and luciferase assay buffer were added to the wells, and their luminescence was determined using a microplate reader. Luciferase activity was expressed as the ratio of promoter-dependent firefly luciferase activity to *β*-galactosidase activity.

### 2.11. Immunocytochemistry

The cells were fixed using 4% paraformaldehyde in PBS for 15 min and permeabilized in 0.1% Triton X-100 and 0.01% Tween 20 for 20 min at room temperature. After blocking the cells with PBS containing 3% bovine serum albumin (BSA), the cells were incubated with anti-FoxO3a (1 : 200; Cell Signaling Technology) antibodies. After being washed thrice, they were incubated with Flamma-594 secondary antibodies (BioActs). Finally, the cells were mounted on glass slides after counterstaining with DAPI and observed under an LSM 700 laser scanning confocal microscope (Zeiss, Jena, Germany) with a C-Apochromat 20x objective. For the measurement of immunofluorescence intensity, images were captured with the same laser power and the mean intensity of fluorescence signals was measured. Data were analyzed using ZEN 2012 Blue (Zeiss) and ImageJ software (National Institutes of Health, Bethesda, MD, USA), under the same processing parameters.

### 2.12. Statistical Analysis

The data are expressed as the mean ± standard error of the mean (SEM). Analyses of differences between two groups were performed using Student's *t*-test. The comparison between multiple groups was performed using one-way analysis of variance (ANOVA), followed by Tukey's multiple comparison test, for which the GraphPad Prism (5.0) (GraphPad, La Jolla, CA, USA) software was used. Statistical significance was considered when the *p* value was less than 0.05.

## 3. Results

### 3.1. Blue Light Inhibits the Proliferation of Human Keratinocytes

**Figure 1:**
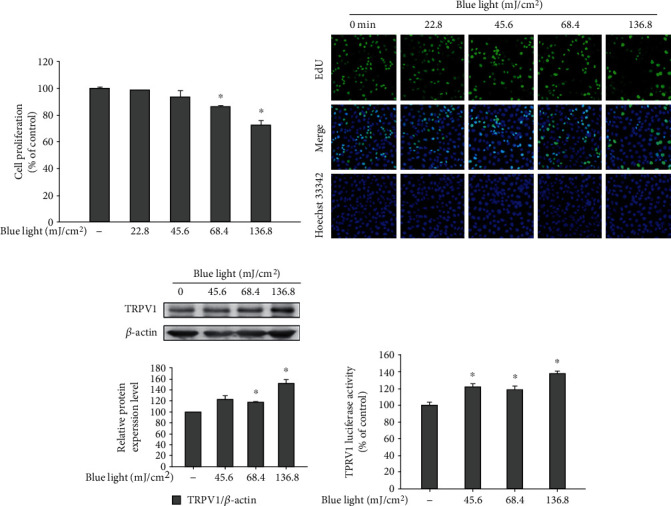
Effects of blue light irradiation on cell proliferation and TRPV1 expression. HaCaT cells were irradiated using blue light with a wavelength of 470–480 nm once daily for three consecutive days (a). On the fourth day, 24 h after the last irradiation, cell proliferation was measured using cell counting kit-8 assay (a) and EdU imaging analysis (b). The protein levels of TRPV1 were also determined by Western blotting analysis (c). HEK293-TRPV1 cells were irradiated using the indicated intensity of blue light with a wavelength of 470–480 nm (d). Twenty-four hours after the irradiation, the cells were harvested and subjected to luciferase reporter assay. Data are presented as the mean ± SEM of four independent experiments. Statistical significance of differences among the groups was assessed by one-way analysis of variance (ANOVA), followed by Tukey's multiple comparison test, using the GraphPad Prism 5 software. ^∗^*p* < 0.05 vs. the control group.

To examine the effects of blue light irradiation on the proliferation of human keratinocytes, cell proliferation assay was performed using HaCaT cells. As shown in Figure 1(a), blue light irradiation reduced the proliferation potential of keratinocytes in a dose-dependent manner.

This was further confirmed by the EdU proliferation assay (Figure 1(b)). In addition, to examine the involvement of TRPV1 in the blue light irradiation-induced reduction of the cell proliferation potential, we performed Western blotting analysis for detecting TRPV1 and the TRPV1-luciferase reporter assay. As shown in Figure 1(c), the protein levels of TRPV1 increased following blue light irradiation. In addition, blue light enhanced the TRPV1-luciferase reporter activity (Figure 1(d)). These data suggest that the effects of blue light on cell proliferation are associated with TRPV1 upregulation.

### 3.2. Blue Light Irradiation Increases the Expression of TRPV1 and Inhibits the EGFR/PI3K/AKT/FoxO3a Pathway

It is known that the activation of TRPV1 induces the degradation of another receptor, EGFR, and then, its downstream AKT signaling pathway is also downregulated [21, 22]. It is also well known that the downregulation of AKT signaling reduces cell proliferation and induces apoptosis [30, 31]. Therefore, we performed Western blotting analysis and real-time PCR analysis to investigate the effects of blue light irradiation on EGFR/PI3K/AKT signaling.

**Figure 2:**
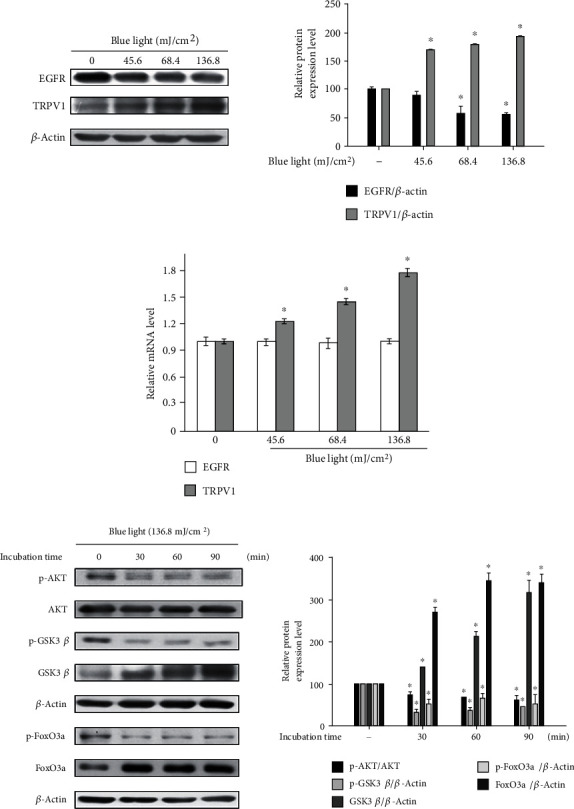
Blue light irradiation inhibits the EGFR/PI3K/AKT signaling pathway by degrading EGFR. (a) HaCaT cells were irradiated using the indicated intensity of blue light with a wavelength of 470–480 nm once daily for three consecutive days. (b) On the fourth day, 24 h after the last irradiation, the cells were harvested and subjected to Western blotting analysis and real-time PCR analysis for analyzing the TRPV1 and EGFR levels. (c) HaCaT cells were irradiated using blue light for 30 min and incubated for the indicated time periods. The cells were harvested immediately after the incubation and subjected to Western blotting analysis for quantifying the phosphorylation levels of AKT and its downstream molecules (GSK3*β* and FoxO3a). ^∗^*p* < 0.05 vs. the control group.

As shown in Figure 2(a), the protein levels of EGFR reduced, while those of TRPV1 increased in a dose-dependent manner. However, although blue light irradiation increased the mRNA levels of the TRPV1 gene, the mRNA levels of the EGFR gene were not affected by blue light irradiation (Figure 2(b)). We also found that the phosphorylation levels of proteins involved in the AKT signaling pathway were reduced. Specifically, as shown in Figure 2(c), the phosphorylation levels of AKT and GSK3*β* were reduced following blue light irradiation. In addition, blue light irradiation reduced the phosphorylation levels of FoxO3a, a downstream molecule of GSK3*β* (Figure 2(c)).

FoxO3a is a transcription factor involved in AKT signaling, and when FoxO3a is phosphorylated by GSK3*β*, it cannot be translocated into the nucleus. In addition, the phosphorylation of FoxO3a was suppressed by blue light irradiation. Therefore, we examined the effects of blue light irradiation on the nuclear translocation of FoxO3a.

**Figure 3:**
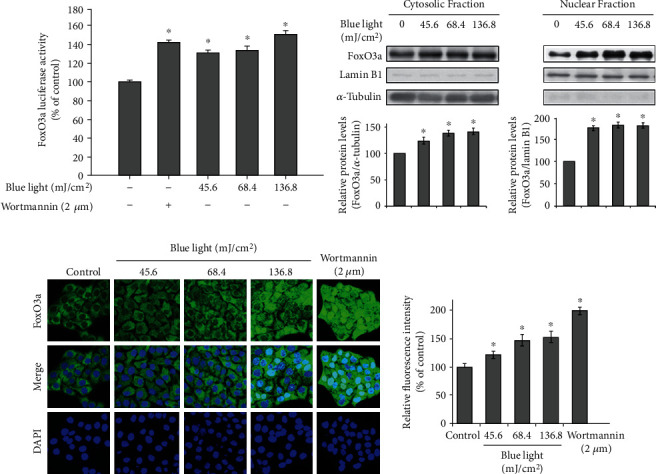
Blue light irradiation promotes the nuclear translocation of FoxO3a. (a) HaCaT cells were cotransfected with the FoxO3a promoter-luciferase reporter and *β*-galactosidase reporter vector using polyethylenimine. After 24 h, the transfected cells were incubated with the indicated intensity of blue light or treated with wortmannin (2 *μ*M), a PI3K inhibitor. After 24 h of incubation, FoxO3a promoter-luciferase reporter activity was measured. In addition, *β*-galactosidase assay was performed to measure transfection efficiency. Data are presented as the mean ± SEM of four independent experiments. Statistical significance of differences among the groups was assessed by one-way analysis of variance (ANOVA), followed by Tukey's multiple comparison test, using the GraphPad Prism 5 software. ^∗^*p* < 0.05 vs. the control group. (b) HaCaT cells were irradiated with the indicated intensity of blue light. After 24 h, the cells were harvested, and the cytoplasmic and nuclear fractions were prepared using the NE-PER™ Nuclear and Cytoplasmic Extraction Reagents, respectively. The nuclear translocation levels of FoxO3a were determined by Western blotting analysis. The densitometric analysis was also performed. ^∗^*p* < 0.05 vs. the control group. (c) HaCaT cells were irradiated with the indicated intensity of blue light or treated with wortmannin. After 24 h, the cells were fixed in 4% formaldehyde solution, and FoxO3a was visualized using rabbit polyclonal antibodies, followed by Alexa 594-labeled antibody (red). Additionally, DAPI (blue) was utilized to visualize the nuclei. The relative fluorescence intensity was also analyzed. ^∗^*p* < 0.05 vs. the control group.

As shown in Figure 3(a), we found that blue light irradiation increased the FoxO3a-response element-luciferase reporter activity. Western blotting analysis showed that the nuclear translocation of FoxO3a occurred in response to blue light irradiation (Figure 3(b)). This effect was also demonstrated by immunocytochemistry (ICC) imaging analysis, which showed that blue light irradiation enhanced the nuclear translocation of FoxO3a (Figure 3(c)). Wortmannin was used as an inhibitor of phosphoinositide 3-kinases (PI3Ks). These data indicate that blue light irradiation inhibits the PI3K/AKT/GSK3*β*/FoxO3a signaling pathway by reducing the EGFR expression.

### 3.3. Knockdown of TRPV1 Attenuates the Antiproliferative Effects of Blue Light

**Figure 4:**
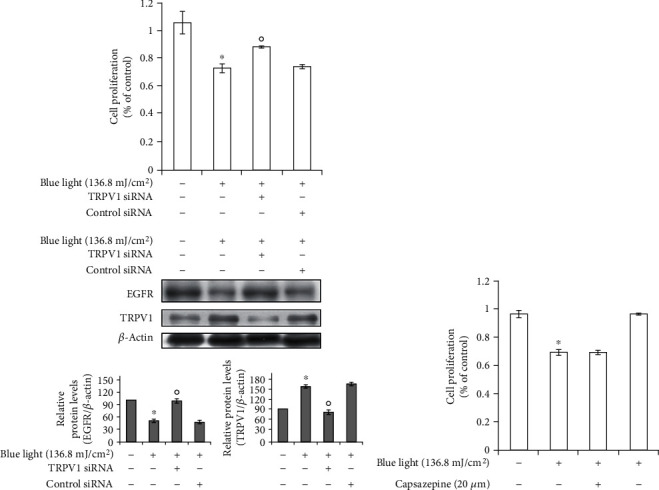
Knockdown of the TRPV1 gene attenuates the effects of blue light on cell proliferation. HaCaT cells were transfected with TRPV1 siRNA, followed by incubation for 16 h. (a) The transfected cells were irradiated using the indicated intensity of blue light with a wavelength of 470–480 nm and further incubated for 24 h. The cells were subjected to cell proliferation testing using cell counting kit-8 assay and Western blotting analysis for assessing the EGFR, TRPV1, or *β*-actin levels. Results were obtained from at least three independent experiments, and the values represent the mean ± SEM. *p* values were obtained by one-way ANOVA. ^∗^*p* < 0.05 vs. the untreated control, ^o^*p* < 0.05 vs. the blue light-irradiated control. (b) HaCaT cells were treated with capsazepine (CPZ), a TRPV1 antagonist. After 30 minutes, they were washed with PBS and irradiated with the indicated intensity of blue light. After 24 hours, the cells were subjected to cell proliferation testing using cell counting kit-8 assay. Results were obtained from at least three independent experiments, and the values represent the mean ± SEM. *p* values were obtained by one-way ANOVA. ^∗^*p* < 0.05 vs. the blue light-irradiated control.

We examined whether TRPV1 is involved in the antiproliferative effects of blue light using small interfering RNA (siRNA) for TRPV1. As shown in Figure 4(a), we found that TRPV1 knockdown attenuated the effects of blue light irradiation on cell proliferation. In addition, blue light-induced reduction of EGFR expression was recovered by TRPV1 knockdown (Figure 4(a)).

These data indicate that the TRPV1-dependent downregulation of EGFR contributes to the antiproliferative effects of blue light. In addition, capsazepine, a TRPV1 antagonist, did not recover the effects of blue light irradiation on cell proliferation (Figure 4(b)). These data suggest that blue light reduces cell proliferation by upregulating TRPV1, not by TRPV1 activation.

### 3.4. Blue Light-Induced ROS Production Is Mediated by TRPV1 Activation

**Figure 5:**
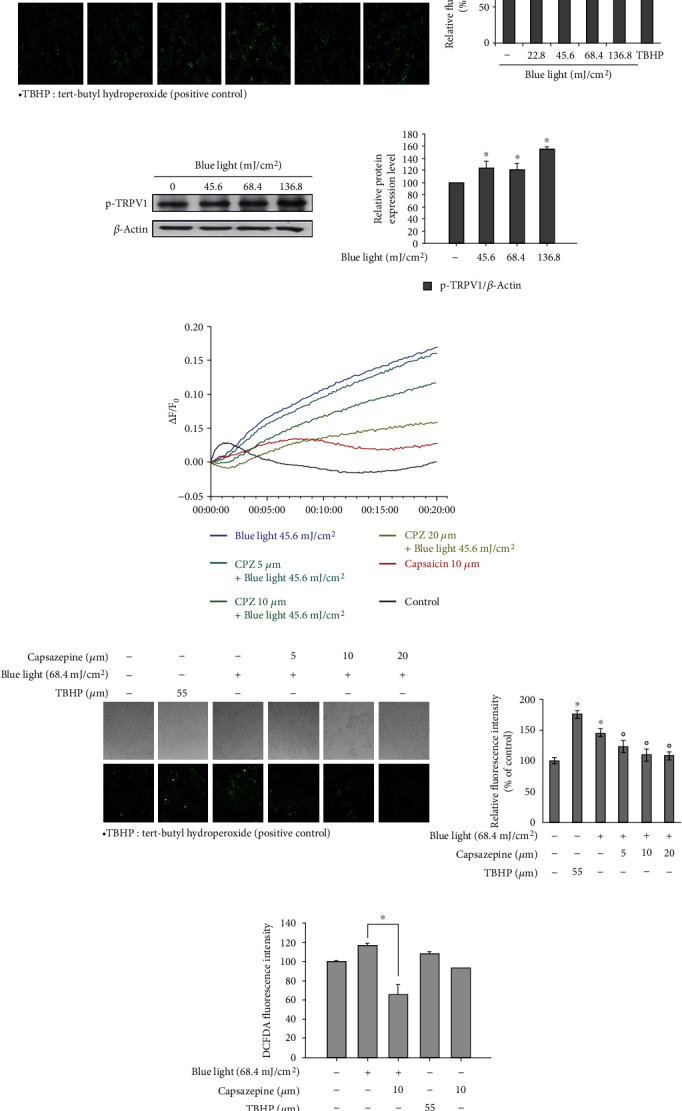
ROS production via TRPV1 activation in response to blue light irradiation. (a) HaCaT cells were irradiated with the indicated intensity of blue light. After 24 hours, they were stained with DCFDA and fluorescent images were recorded. TBHP: tert-butyl hydroperoxide. The relative fluorescence intensity was also analyzed. ^∗^*p* < 0.05 vs. the control group. (b) HaCaT cells were irradiated using blue light with a wavelength of 470–480 nm once daily for three consecutive days. On the fourth day, 24 h after the last irradiation, the phosphorylation levels of TRPV1 were determined by Western blotting analysis. (c) Calcium influx into HaCaT cells was measured by the Fluo-4 Direct assay. After staining with the Fluo-4 reagent, the HaCaT cells were treated with capsazepine, a TRPV1 antagonist. After 30 minutes, the cells were washed with PBS and irradiated with the indicated intensity of blue light. Their fluorescence intensities were measured immediately after the irradiation. (d) HaCaT cells were treated with capsazepine (CPZ), a TRPV1 antagonist. After 30 minutes, they were washed with PBS and irradiated with the indicated intensity of blue light. After 24 hours, they were stained with DCFDA and fluorescent images were recorded. Data are presented as the mean ± SEM of four independent experiments. Statistical significance of differences among the groups was assessed by one-way analysis of variance (ANOVA), followed by Tukey's multiple comparison test, using the GraphPad Prism 5 software. ^∗^*p* < 0.05 vs. the control group. (e) DCFDA-stained cells were also analyzed by measuring their fluorescence using a microplate reader at an Ex/Em of 485/535 nm. The relative fluorescence intensity was also analyzed. ^∗^*p* < 0.05 vs. the untreated control, ^o^*p* < 0.05 vs. the blue light-irradiated control.

The effects of blue light on ROS production have been already reported [29, 32]. In this study, as shown in Figure 5(a), we also assessed the ROS-producing effect of blue light using DCFDA imaging analysis. Tert-butyl hydroperoxide (TBHP) was used to induce cellular ROS production as a positive control.

To further examine the involvement of TRPV1 in the blue light-induced ROS production, Western blotting analysis for detecting phosphorylated TRPV1 and calcium influx analysis were performed. As shown in Figure 5(b), phosphorylation levels of TRPV1 increased following blue light irradiation in a dose-dependent manner. While the levels of calcium increased following blue light irradiation, capsazepine, a TRPV1 antagonist, reversed the effects of blue light on calcium influx (Figure 5(c)). In addition, capsazepine reduced the blue light-induced production of ROS (Figures 5(d) and 5(e)). These results indicate that blue light-induced ROS production is dependent on TRPV1.

### 3.5. Blue Light Increases TNF-*α* Secretion via the NF-*κ*B and AP-1 Signaling Pathways

It is well known that ROS induces inflammatory reactions [33]. Therefore, we examined the effects of blue light irradiation on the production of proinflammatory cytokines.

**Figure 6:**
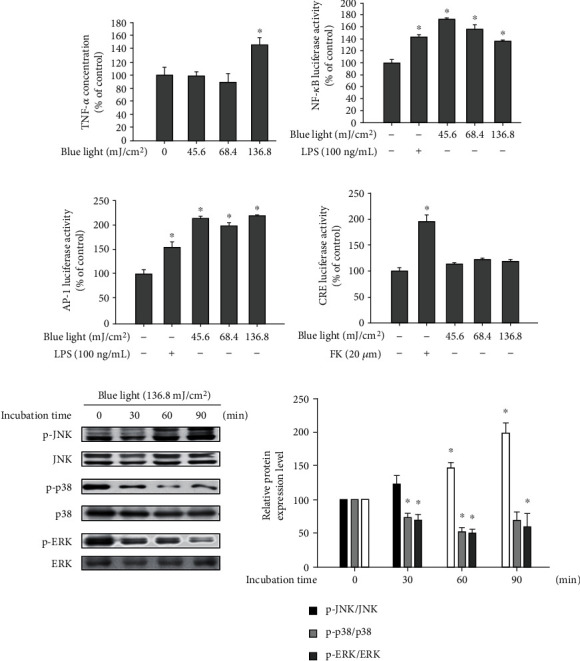
Blue light upregulates proinflammatory cytokine production via NF-*κ*B and AP-1 activation. (a) HaCaT cells were irradiated using the indicated intensity of blue light with a wavelength of 470–480 nm once daily for three consecutive days. On the fourth day, 24 h after the last irradiation, the cells were harvested and subjected to ELISA for analyzing the TNF-*α* levels. Data are presented as the mean ± SEM of four independent experiments. Statistical significance of differences among the groups was assessed by one-way analysis of variance (ANOVA), followed by Tukey's multiple comparison test, using the GraphPad Prism 5 software. ^∗^*p* < 0.05 vs. the control group. (b–d) HaCaT cells were cotransfected with the NF-*κ*B, AP-1, or CRE promoter-luciferase reporters and *β*-galactosidase reporter vector using polyethylenimine. After 24 h, the transfected cells were irradiated with the indicated intensity of blue light. Twenty-four hours after the irradiation, the cells were harvested and subjected to luciferase reporter assay. Data are presented as the mean ± SEM of four independent experiments. Statistical significance of differences among the groups was assessed by one-way analysis of variance (ANOVA), followed by Tukey's multiple comparison test, using the GraphPad Prism 5 software. ^∗^*p* < 0.05 vs. the control group. (e) Cells were irradiated with blue light and incubated for the indicated time periods. The cells were harvested immediately after the incubation, and the protein levels of MAPKs and their phosphorylated forms were detected by Western blotting analysis.

As shown in Figure 6(a), blue light irradiation increased the production of TNF-*α*. To elucidate the mechanisms underlying the blue light-induced production of TNF-*α*, activator protein 1 (AP-1), nuclear factor *κ*B (NF-*κ*B), and cyclic response element (CRE) promoter-luciferase assays were performed. We found that while blue light had no effect on CRE reporter activity, the NF-*κ*B and AP-1 reporters were activated by blue light irradiation (Figures 6(b)–6(d)). LPS was used as a positive control in the NF-*κ*B and AP-1 reporter assays, and forskolin was used to stimulate adenylate cyclase in the CRE reporter assay. In addition, among mitogen-activated protein kinases (MAPKs), blue light irradiation increased the phosphorylation levels of JNK. However, phosphorylation levels of p38 MAPK and p44/42 MAPK were reduced by blue light irradiation (Figure 6(e)). Collectively, these data suggest that the blue light-induced production of TNF-*α* is mediated via the activation of JNK and NF-*κ*B.

### 3.6. TRPV1 Is Involved in Blue Light-Induced TNF-*α* Production

**Figure 7:**
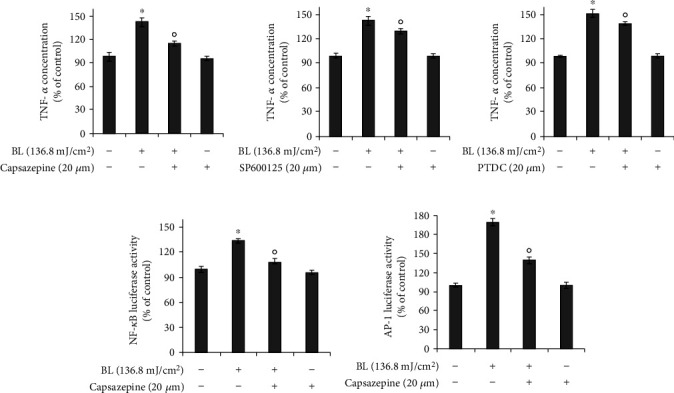
Blue light-induced production of TNF-*α* is dependent on TRPV1-mediated signaling. (a) HaCaT cells were irradiated using the indicated intensity of blue light with a wavelength of 470–480 nm and further incubated for 24 h in the presence of capsazepine, SP600125 (JNK inhibitor), or pyrrolidine dithiocarbamate (PDTC). The cells were harvested and subjected to ELISA for examining the TNF-*α* levels. Data are presented as the mean ± SEM of four independent experiments. (b) HaCaT cells were transfected with the NF-*κ*B or AP-1 reporters and the *β*-galactosidase reporter vector using polyethylenimine, followed by incubation for 16 h. The transfected cells were irradiated using the indicated intensity of blue light with a wavelength of 470–480 nm and further incubated for 24 h in the presence of capsazepine, SP600125, or pyrrolidine dithiocarbamate (PDTC). The cells were harvested and subjected to luciferase reporter assay. Results were obtained from at least three independent experiments, and the values represent the mean ± SEM. Statistical significance of differences among the groups was assessed by one-way analysis of variance (ANOVA), followed by Tukey's multiple comparison test, using the GraphPad Prism 5 software. ^∗^*p* < 0.05 vs. the control group, ^o^*p* < 0.05 vs. the blue light-irradiated control. PDTC: pyrrolidine dithiocarbamate; BL: blue light irradiation.

To investigate the involvement of TRPV1 in blue light-induced TNF-*α* production, we performed luciferase reporter assay for examining the NF-*κ*B and AP-1 reporters and ELISA for detecting TNF-*α* using capsazepine, a TRPV1 antagonist. As shown in Figure 7(a), we found that the blue light irradiation-induced TNF-*α* production was attenuated by capsazepine treatment. In addition, SP600125 (a JNK inhibitor) or PDTC (a NF-*κ*B inhibitor) reduced the blue light irradiation effect on the TNF-*α* production. Similarly, blue light-induced activation of the NF-*κ*B and AP-1 reporters was also attenuated by capsazepine treatment (Figure 7(b)).

These data indicate that TRPV1 is involved in the blue light-induced production of proinflammatory cytokines and TRPV1 operates upstream of AP-1 or NF-*κ*B signaling in the TNF-*α* production.

**Figure 8:**
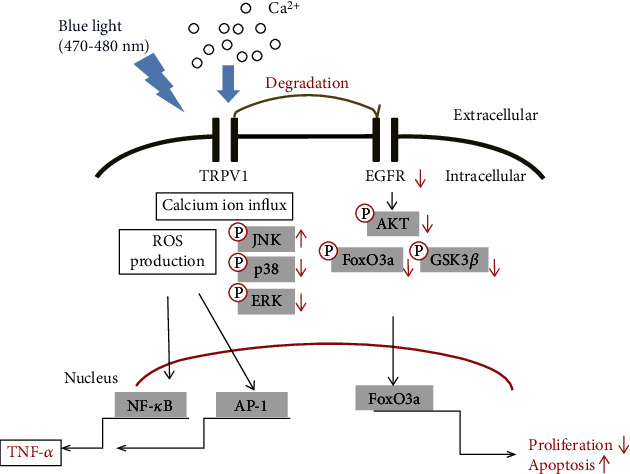
Mechanisms of the effects of blue light irradiation on cell survival and the production of ROS and cytokines in human keratinocytes.

In addition, the mechanisms underlying the effects of blue light on keratinocytes are shown in Figure 8.

## 4. Discussion

The human body is always exposed to sunlight, and the skin plays a primary role in protecting the body from strong sunlight. Among the various constituents of sunlight, many studies have reported the response of skin to, and the protection against, UV light. However, research studies on visible light, which is the major constituent of sunlight, has only begun recently. Therefore, in this study, we examined the effects of blue light irradiation on keratinocytes and the mechanisms underlying these effects.

In this study, the TRPV1 receptor was considered a potential target molecule of blue light. TRPV1 is known to be closely related to various aspects of skin biology such as the survival, proliferation, apoptosis, and proinflammatory responses of skin cells [9, 10, 13]. However, since there is no systematic information about the relationship between the effects of blue light on the skin and TRPV1, we investigated whether TRPV1 is involved in the effects of blue light on the skin. We found that blue light irradiation induced TRPV1 activation in keratinocytes, thereby inducing ROS production. The produced ROS contributed to the production of the proinflammatory cytokine TNF-*α* by activating NF-*κ*B and AP-1 signaling. In addition, we demonstrated that blue light upregulates the expression of TRPV1, which induces EGFR degradation. TRPV1-dependent reduction of EGFR levels suppressed AKT/GSK3*β*/FoxO3a signaling, leading to reduced cell proliferation.

Previous studies have shown that ROS sensitizes TRPV1, and an increase in the cellular calcium ion concentration induces ROS synthesis [20, 34]. However, the relationship between TRPV1 and ROS formation has not yet been clearly elucidated. In this study, we found that blue light irradiation activated TRPV1, leading to increased calcium influx and ROS production. Blue light-induced calcium influx was also attenuated by treatment with capsazepine, a TRPV1 antagonist. In addition, we demonstrated that blue light-induced ROS production was reduced by capsazepine treatment, indicating that blue light irradiation induces ROS production by activating TRPV1.

It is already known that ROS activates the AP-1 and NF-*κ*B promoters [35]. These two transcription factors contribute to the production of proinflammatory cytokines. Similarly, in this study, blue light irradiation was shown to increase ROS production. We also found that blue light enhanced the AP-1 and NF-*κ*B promoter activities, but not the CRE promoter activity. As expected, TNF-*α* secretion was increased by blue light irradiation. These data suggest that the induction of the AP-1 and NF-*κ*B signaling pathways by ROS may contribute to the production of proinflammatory cytokines. In addition, the increased production of TNF-*α* was attenuated by capsazepine treatment. Collectively, these findings suggest that TRPV1-dependent ROS production could induce the production of proinflammatory cytokines.

TRPV1 affects cellular programming by inducing calcium ion influx or the degradation of EGFR [21, 22]. TRPV1 induces the degradation of another receptor, EGFR, and then, its downstream AKT signaling pathway is also downregulated [21, 22]. It is also well known that the downregulation of AKT signaling reduces cell proliferation and induces apoptosis [30, 31]. In this study, we found that blue light increased the expression of the TRPV1 gene. However, although blue light decreased the protein levels of EGFR, it did not affect its mRNA levels. In addition, blue light suppressed its downstream AKT/GSK3*β* signaling pathway. FoxO3a, a downstream molecule of AKT/GSK3*β* signaling [36–39], has been reported to be involved in both antioxidant activity and reduced cell proliferation. In this study, we found that blue light irradiation induced the nuclear translocation of FoxO3a. The knockdown of TRPV1 using TRPV1 siRNA was found to attenuate the inhibitory effects of blue light irradiation on cell proliferation. These data suggest that blue light-induced upregulation of TRPV1 promotes the degradation of EGFR, leading to a reduction of cell proliferation. Specifically, TRPV1-induced degradation of EGFR inhibits the EGFR/AKT/GSK3*β*/FoxO3a pathway, resulting in the blue light-induced antiproliferation effects.

When the proliferation of keratinocytes is abnormally regulated, a variety of diseases can be induced [40]. One such representative disease is psoriasis. Under conditions of normal skin homeostasis, keratinocytes accumulate on the surface at the end of their differentiation stage to form a skin barrier, after which their differentiation is turned off. In psoriasis, the hyperproliferation of epidermal keratinocytes is induced; thus, these cells have a faster turnover cycle than normal cells [41–43]. This causes red or pale pink lesions covered with silvery scales of dead skin cells [42]. These are called plaques and are sometimes accompanied by itching or a sore feeling. Keratinocyte carcinoma also involves the excessive proliferation of keratinocytes and is one of the most common skin cancers among people who have light skin [44]. In our study, we found that blue light irradiation attenuated the proliferation potential of keratinocytes. Therefore, these data suggest that blue light irradiation may serve as a useful approach for treating hyperproliferative human dermatoses such as psoriasis or keratinocyte-derived skin tumors.

## 5. Conclusions

We demonstrated that blue light increases the calcium ion influx through the activation of TRPV1 in keratinocytes, leading to the production of ROS and proinflammatory cytokines. In addition, blue light-induced upregulation of TRPV1 reduced the protein levels of EGFR and then suppressed AKT/GSK3*β*/FoxO3a signaling, consequently resulting in reduced cell proliferation. Furthermore, these data suggest that the blue light-induced suppression of keratinocyte proliferation could become a useful adjunct approach for treating hyperproliferative human dermatoses such as psoriasis or keratinocyte-derived skin tumors.

## Data Availability

The data used to support the findings of this study are available from the corresponding authors upon request.

## Abbreviations

TRPV1: Transient receptor potential vanilloid 1
EGFR: Epidermal growth factor receptor
ROS: Reactive oxygen species
TNF-*α*: Tumor necrosis factor-*α*.

## References

[1] Rawlings A., Harding C. (2004). Moisturization and skin barrier function. *Dermatologic Therapy*.

[2] Zhang H., Hou W., Henrot L. (2015). Modelling epidermis homoeostasis and psoriasis pathogenesis. *Journal of The Royal Society Interface*.

[3] Proksch E., Fölster-Holst R., Jensen J.-M. (2006). Skin barrier function, epidermal proliferation and differentiation in eczema. *Journal of Dermatological Science*.

[4] Weinstein G. D., McCullough J. L., Ross P. (1984). Cell proliferation in normal epidermis. *The Journal of Investigative Dermatology*.

[5] Milstone L. M. (2004). Epidermal desquamation. *Journal of Dermatological Science*.

[6] Eckert R. L., Rorke E. A. (1989). Molecular biology of keratinocyte differentiation. *Environmental Health Perspectives*.

[7] Elsholz F., Harteneck C., Muller W., Friedland K. (2014). Calcium--a central regulator of keratinocyte differentiation in health and disease. *European Journal of Dermatology*.

[8] Bikle D. D., Xie Z., Tu C. L. (2014). Calcium regulation of keratinocyte differentiation. *Expert Review of Endocrinology and Metabolism*.

[9] Tóth B. I., Oláh A., Szöllősi A. G., Bíró T. (2014). TRP channels in the skin. *British Journal of Pharmacology*.

[10] Caterina M. J., Pang Z. (2016). TRP channels in skin biology and pathophysiology. *Pharmaceuticals*.

[11] Zheng J. (2013). Molecular mechanism of TRP channels. *Comprehensive Physiology*.

[12] Liang J., Xiao G., Ji W. (2011). Capsaicin induces reflex scratching in inflamed skin. *Pharmacology*.

[13] Lee Y. M., Kang S. M., Chung J. H. (2012). The role of TRPV1 channel in aged human skin. *Journal of Dermatological Science*.

[14] Lee Y. M., Kang S. M., Lee S. R. (2011). Inhibitory effects of TRPV1 blocker on UV-induced responses in the hairless mice. *Archives of Dermatological Research*.

[15] Li W. H., Lee Y. M., Kim J. Y. (2007). Transient receptor potential vanilloid-1 mediates heat-shock-induced matrix metalloproteinase-1 expression in human epidermal keratinocytes. *The Journal of Investigative Dermatology*.

[16] Graham D. M., Huang L., Robinson K. R., Messerli M. A. (2013). Epidermal keratinocyte polarity and motility require Ca2+ influx through TRPV1. *Journal of Cell Science*.

[17] Devesa I., Planells-Cases R., Fernández-Ballester G., González-Ros J. M., Ferrer-Montiel A., Fernández-Carvajal A. (2011). Role of the transient receptor potential vanilloid 1 in inflammation and sepsis. *Journal of Inflammation Research*.

[18] Kueper T., Krohn M., Haustedt L. O., Hatt H., Schmaus G., Vielhaber G. (2010). Inhibition of TRPV1 for the treatment of sensitive skin. *Experimental Dermatology*.

[19] Lee Y. M., Kim Y. K., Kim K. H., Park S. J., Kim S. J., Chung J. H. (2009). A novel role for the TRPV1 channel in UV-induced matrix metalloproteinase (MMP)-1 expression in HaCaT cells. *Journal of Cellular Physiology*.

[20] Hu F., Sun W. W., Zhao X. T., Cui Z. J., Yang W. X. (2008). TRPV1 mediates cell death in rat synovial fibroblasts through calcium entry-dependent ROS production and mitochondrial depolarization. *Biochemical and Biophysical Research Communications*.

[21] Bode A. M., Cho Y. Y., Zheng D. (2009). Transient receptor potential type vanilloid 1 suppresses skin carcinogenesis. *Cancer Research*.

[22] Li S., Bode A. M., Zhu F. (2011). TRPV1-antagonist AMG9810 promotes mouse skin tumorigenesis through EGFR/Akt signaling. *Carcinogenesis*.

[23] Nakamura M., Kuse Y., Tsuruma K., Shimazawa M., Hara H. (2017). The involvement of the oxidative stress in murine blue LED light-induced retinal damage model. *Biological & Pharmaceutical Bulletin*.

[24] Krigel A., Berdugo M., Picard E. (2016). Light-induced retinal damage using different light sources, protocols and rat strains reveals LED phototoxicity. *Neuroscience*.

[25] Dayang W., Dongbo P. (2018). Taurine reduces blue light-induced retinal neuronal cell apoptosis in vitro. *Cutaneous and Ocular Toxicology*.

[26] Lee Y. M., Kim Y. K., Chung J. H. (2009). Increased expression of TRPV1 channel in intrinsically aged and photoaged human skin in vivo. *Experimental Dermatology*.

[27] Kang S. M., Han S., Oh J. H. (2017). A synthetic peptide blocking TRPV1 activation inhibits UV-induced skin responses. *Journal of Dermatological Science*.

[28] Liebmann J., Born M., Kolb-Bachofen V. (2010). Blue-light irradiation regulates proliferation and differentiation in human skin cells. *The Journal of Investigative Dermatology*.

[29] Nakashima Y., Ohta S., Wolf A. M. (2017). Blue light-induced oxidative stress in live skin. *Free Radical Biology & Medicine*.

[30] Cantley L. C. (2002). The phosphoinositide 3-kinase pathway. *Science*.

[31] Yu J. S., Cui W. (2016). Proliferation, survival and metabolism: the role of PI3K/AKT/mTOR signalling in pluripotency and cell fate determination. *Development*.

[32] Godley B. F., Shamsi F. A., Liang F. Q., Jarrett S. G., Davies S., Boulton M. (2005). Blue light induces mitochondrial DNA damage and free radical production in epithelial cells. *The Journal of Biological Chemistry*.

[33] Mittal M., Siddiqui M. R., Tran K., Reddy S. P., Malik A. B. (2014). Reactive oxygen species in inflammation and tissue injury. *Antioxidants & Redox Signaling*.

[34] Kishimoto E., Naito Y., Handa O. (2011). Oxidative stress-induced posttranslational modification of TRPV1 expressed in esophageal epithelial cells. *American Journal of Physiology. Gastrointestinal and Liver Physiology*.

[35] Dhar A., Young M. R., Colburn N. H. (2002). The role of AP-1, NF-kappaB and ROS/NOS in skin carcinogenesis: the JB6 model is predictive. *Molecular and Cellular Biochemistry*.

[36] Liu Y., Ao X., Ding W. (2018). Critical role of FOXO3a in carcinogenesis. *Molecular Cancer*.

[37] Qian K., Wang G., Cao R. (2016). Capsaicin suppresses cell proliferation, induces cell cycle arrest and ROS production in bladder cancer cells through FOXO3a-mediated pathways. *Molecules*.

[38] Klotz L.-O., Sánchez-Ramos C., Prieto-Arroyo I., Urbánek P., Steinbrenner H., Monsalve M. (2015). Redox regulation of FoxO transcription factors. *Redox Biology*.

[39] Zhang X., Tang N., Hadden T. J., Rishi A. K. (2011). Akt, FoxO and regulation of apoptosis. *Biochimica et Biophysica Acta*.

[40] Franssen M. E. J., Boezeman J. B. M., van de Kerkhof P. C. M., van Erp P. E. J. (2004). Monitoring hyperproliferative disorders in human skin: flow cytometry of changing cytokeratin expression. *Cytometry. Part B, Clinical Cytometry*.

[41] Ziboh V. A. (1988). Psoriasis: hyperproliferative/inflammatory skin disorder. *Drug Development Research*.

[42] Boehncke W. H., Schon M. P. (2015). Psoriasis. *Lancet*.

[43] Valdimarsson H., Bake B. S., Jónsdótdr I., Fry L. (1986). Psoriasis: a disease of abnormal keratinocyte proliferation induced by T lymphocytes. *Immunology Today*.

[44] Nagarajan P., Asgari M. M., Green A. C. (2019). Keratinocyte carcinomas: current concepts and future research priorities. *Clinical Cancer Research*.

